# Association between atherogenic index of plasma and future cardiovascular disease risk in middle-aged and elderly individuals with cardiovascular-kidney-metabolic syndrome stage 0-3

**DOI:** 10.3389/fendo.2025.1540241

**Published:** 2025-03-14

**Authors:** Ya Lin, Xiaodong Lv, Ce Shi, Ting Wang, Zehao Jin, Qiangsong Jin, Chao Gu

**Affiliations:** ^1^ Department of Pulmonary and Critical Care Medicine, The First Affiliated Hospital of Wenzhou Medical University, Wenzhou, Zhejiang, China; ^2^ Department of Respiratory Medicine, The First Hospital of Jiaxing (Affiliated Hospital of Jiaxing University), Jiaxing, Zhejiang, China; ^3^ School of Digital Economics, Wenzhou Vocational College of Science and Technology, Wenzhou, Zhejiang, China; ^4^ Department of Ultrasound, Shaoxing People’s Hospital, Shaoxing, Zhejiang, China; ^5^ School of Medicine and Health, Technical University of Munich, Munich, Germany; ^6^ Department of Cardiology, Affiliated Jinhua Hospital, Zhejiang University School of Medicine, Jinhua, Zhejiang, China

**Keywords:** atherogenic index of plasma, cardiovascular-kidney-metabolic syndrome, cardiovascular disease, CHARLS, K-mean clustering analysis

## Abstract

**Background:**

Cardiovascular disease (CVD) is strongly correlated with plasma atherogenic index (AIP); however, there is limited literature exploring the association between trajectories of change in AIP and the risk of CVD. This study aimed to investigate whether changes in AIP are associated with CVD in individuals with cardiovascular-kidney-metabolic (CKM) syndrome stage 0-3.

**Methods:**

Data were sourced from the China Health and Retirement Longitudinal Study (CHARLS), aimed to compile high-quality microdata on individuals and households aged 45 and older in China. Change in AIP from 2012 to 2015 were classified employing K-means clustering analysis. Logistic regressions were employed to assess the association between different AIP change clusters and cumulative AIP and CVD incidence. Additionally, restricted cubic spline (RCS) regression was conducted to further evaluate the underlying linear relationship between cumulative AIP and CVD. Subgroup analyses were applied to verify the influence of confounding variables on the relationship between AIP and CVD. Weighted quantile sum (WGS) regressions were utilized to offer a comprehensive assessment of the overall effect.

**Results:**

Out of 4,525 participants, 578 (12.77%) ultimately developed CVD within three years. Compared to cluster 1, which served as the best control for AIP, the odds ratio (OR) was 1.29 (1.02-1.62) for cluster 2, 1.33 (1.04-1.71) for cluster 3 and 1.35 (0.98-1.85) for cluster 4 after adjusting for several confounding variables. Categorizing the cumulative AIP into quartiles revealed an ascending trend (P for trend = 0.014). RCS regression disclosed a linear relationship between cumulative AIP and CVD. Further subgroup analyses revealed variations in these correlations modified by gender and Hukou status. WQS regression analysis highlighted the significance of triglyceride in the pathogenesis of CVD.

**Conclusions:**

Significant changes in AIP are independently associated with the elevated risk of CVD in adults aged > 45 with CKM syndrome stage 0-3. Monitoring long-term fluctuations in AIP may aid in the early identification of individuals at high risk for CVD.

## Introduction

Cardiovascular diseases (CVD), encompassing conditions such as heart disease and stroke, are major contributors to global morbidity and mortality (1). By 2021, the overall prevalence of CVD had reached 523 million, a significant rise from the 271 million reported in 1990 (2). The death rate of CVD has been on a steady decline over the past few decades; however, there has been an observably ascending trend in CVD mortality since the onset of the COVID-19 pandemic, which has exerted a persistent influence on the ongoing disease burden and its relevant strain on health systems (3).

Cardiovascular-kidney-metabolic (CKM) syndrome is a complex systemic condition manifested as interrelated pathophysiological mechanisms involving metabolic risk factors, chronic kidney disease (CKD) and cardiovascular dysfunction, resulting in multi-organ impairment and an elevated risk of adverse cardiovascular events (4). Epidemiological studies have demonstrated that progression through CKM syndrome stages is closely associated with an increase in both relative and absolute risks of CVD (5). Moreover, the clinical impact of CKM syndrome is incommensurately linked to CVD (6). Consequently, it is imperative that early identification and comprehensive management of early stage of CKM syndrome will contribute to the prevention of the onset and progression of CVD.

The atherogenic index of plasma (AIP) has emerged as a newly identified lipid-metabolism-associated biomarker for plasma atherosclerosis (7). Extensive studies have demonstrated that AIP is correlated with multiple hazard for CVD, including hypertension (8), obesity (9), diabetes mellitus (DM) (10, 11), and metabolic syndrome (MetS) (12). Increasing evidence supports that AIP is a reliable biomarker and a potentially valuable tool for clinicians in predicting the onset and prognosis of CVD (13–16).

A limited number of studies have probed the link between changes in the AIP and the incidence of CVD in the existing research (17–19). The inherent limitation of previous studies was that the AIP was typically appraised at a single time point, failing to capture the dynamic relationship between longitudinal fluctuations in AIP and CVD risk over time, which may not adequately reflect the impact of prolonged exposure. Longitudinal studies examining the effects of repeated AIP measurements on CVD risk remain limited. Thus, it remains uncertain whether controlling AIP further influences the presence of CVD in participants with CKM syndrome. The objective of our longitudinal study is to ascertain the association between AIP controlling level and the danger of CVD in individuals classified with CKM syndrome stage 0 to 3.

## Materials and methods

### Study population and exclusion criteria

The China Health and Retirement Longitudinal Study (CHARLS) is the data source of this study, the first nationally representative longitudinal survey targeting Chinese middle-aged and elderly populations. The baseline survey (Wave 1) was carried out between 2011 and 2012, subsequent to Wave 2 in 2013, Wave 3 in 2015, and Wave 4 in 2018 and the multistage probability proportional to size sampling strategy was adopted (20).

Ethical approval for all the CHARLS waves was granted by the Institutional Review Board (IRB) at Peking University - main household survey (IRB00001052-11015) and biomarker collection (IRB00001052-11014).

The baseline survey collected a total of 17,705 participants at Wave 1, covering 150 counties/450 villages/10,000 households. Comprehensive data on demographics, health status, physical measurements, and laboratory parameters were collected. Ultimately, 4,525 participants were recruited for the final analysis after applying exclusion criteria (**Supplementary Figure 1**). The exclusion criteria were described below (1): lack of relevant information regarding the diagnosis of CKM syndrome (2); age under 45 years (3); absence of TG or HDL-C testing data at Wave 1 or Wave 3 (4); individuals who self-reported CVD incidents prior to 2015 (5); individuals who did not provide incomplete information on the CVD incidence during the three-year follow-up period.

### Definition of CKM syndrome stages

The stages of CKM syndrome, ranging from 0 to 3, were categorized on the basis of the cardiovascular-kidney-metabolic health presidential advisory from the American Heart Association. The classification was as following: stage 0, no risk factors of CKM present; stage 1, excess or abnormal adiposity; stage 2, metabolic risk factors or moderate-risk to high-risk CKD; and stage 3, subclinical CVD overlapping with CKM risk factors (21). Within this framework, very high-risk CKD (stage G4 or G5) and advanced predicted 10-year risk of CVD based on the Framingham risk score were considered equivalent to those with subclinical CVD (22). The modified Modification of Diet in Renal Disease equation was applied to obtain estimated glomerular filtration ratio (eGFR) (23), which was divided into CKD stages according to Kidney Disease Improving Global Outcomes guidelines (24).

### Definitions of outcomes

CVD was designated as the dominating outcome of the study, whereas heart disease and stroke were the secondary outcomes. The question “Have you been diagnosed with heart attack, coronary heart disease, angina, congestive heart failure, or other heart problems by a doctor?” identified whether participants were the incidence of heart disease; the question “Have you been diagnosed with stroke by a doctor?” indicated whether participants were the presence of stroke. The diagnosis of CVD was in the light of the participants’ self-reported medical histories concerning heart disease or stroke.

### Assessment of AIP and cumulative AIP

The TG and HDL-C levels were measured through the tests of blood sample conducted during Wave 1 and Wave 3, with both parameters expressed in mmol/L. AIP and cumulative AIP were gained using the following equations:


AIP= log (TG / HDL−C)

$$\text{Cumulative AIP}=\left(AIP_{2012}\;+\;AIP_{2015}\right)\;/\;2\;\times \text{ time }\left(2015\;–\;2012\right)$$

### Assessment of covariates

Demographic covariates included age, gender, marital status, Hukou status, educational level, smoking status and drinking status. Basic physical measurements comprised of systolic blood pressure, diastolic blood pressure, body mass index and waist circumference. Comorbidities and complications included hypertension, diabetes, dyslipidemia, MetS and kidney disease. Medication history included the use of antihypertensive, antidiabetic, and lipid-lowering drugs. Laboratory examination included white blood cell (WBC), hemoglobin (Hb), platelets, fasting blood glucose (FBG), glycated hemoglobin A1C, total cholesterol (TC), TG, HDL-C, low-density lipoprotein cholesterol (LDL-C), C-reactive protein (CRP), blood urea nitrogen, serum creatinine and eGFR.

### Clustering of the changes in AIP

**Figure 1:**
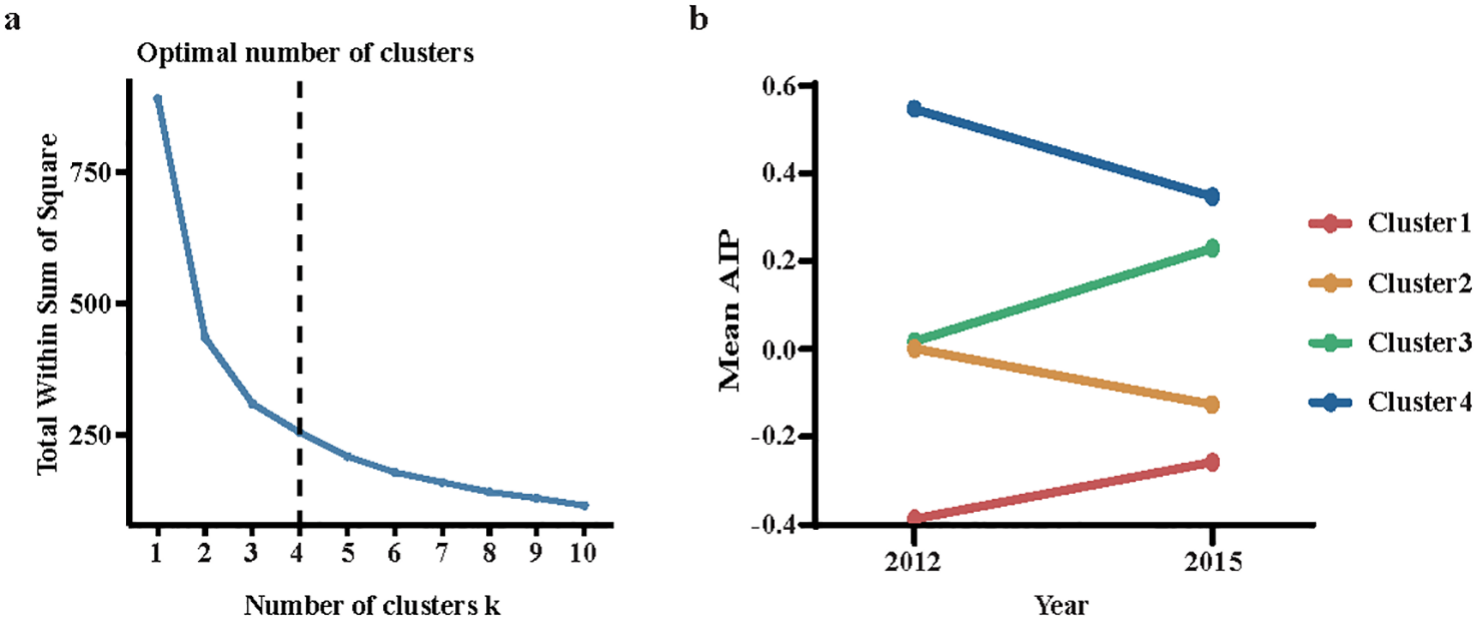
Clustering of the change in the AIP from 2012 to 2015. **(a)** The elbow method for determining the optimal number of clusters; **(b)** Data visualization for the clusters of the change in the AIP. AIP, atherogenic index of plasma.

K-means clustering analysis is a commonly used unsupervised learning algorithm that iteratively solves problems based on distance measurement (25). Using the elbow method, the dataset was categorized into four distinct clusters: Cluster 1, with AIP values ranging from -0.3467 to -0.2174, representing consistently low AIP levels; Cluster 2, with AIP values from 0.0409 to -0.0868, indicating moderate AIP levels that decrease over time; Cluster 3, with AIP values between 0.0576 and 0.2696, reflecting moderate AIP levels that increase over time; and Cluster 4, with AIP values from 0.5863 to 0.3866, indicating the highest AIP levels with a declining trend (**Figure 1**).

### Statistical analysis

The data distribution of all continuous variables was assessed employing the Shapiro-Wilk test, which indicated that all data were non-normally distributed. The continuous variables were presented as median and interquartile range (IQR). The categorical variables were expressed as counts and percentages. To compare statistical differences across the AIP clusters, either the Kruskal-Wallis test or Fisher’s exact test was used.

Multivariable logistic regression analyses were utilized to illuminate the relationships between AIP and CVD across four models; and all the results were summarized as odds ratio (OR) and 95% confidence interval (CI). The regression models were concluded as follows: Crude model I (unadjusted); adjusted model II (age and gender); adjusted model III (age, gender, marital status, Hukou status, educational level, smoking status, and drinking status); and adjusted model IV (age, gender, marital status, Hukou status, educational level, smoking status, drinking status, WBC, Hb, platelets and CRP). The potential dose–response relationships between cumulative AIP and CVD were inspected by restricted cubic spline (RCS) models, which were adjusting for same covariates as in Model IV. Additionally, subgroup analyses were performed to estimate the impact of confounding variables on the association between AIP and CVD. The weighted quantile sum (WQS) regression models were implemented to quantify the relative contributions of blood lipid and glucose levels to the overall effect.

Statistical analyses were performed using R software version 4.3.0; and statistical significance was set at p < 0.05.

## Results

### Baseline characteristics of participants

In this study, a total of 4,525 participants diagnosed with CKM stage 0-3 were enrolled, with a median age of 58 years, and 2084 males (46.06%). The median AIP was -0.04 (-0.24, 0.19) in 2012 and -0.01 (-0.18, 0.20) in 2015, with a median cumulative AIP of -0.06 (-0.58, 0.52). Over the three-year follow-up period, the incidence of CVD, heart disease and stroke were observed to be 8.13%, 5.44% and 12.77%, respectively. On the basis of the clustering analysis, the baseline characteristics in each cluster are detailed in **Table 1**.

**Table 1:** Baseline characteristics of participants by K-means clustering analysis.

Characteristics	Overall (n = 4,525)	Change in the AIP				P value
		Cluster 1 (n = 1,380)	Cluster 2 (n = 1,555)	Cluster 3 (n = 1,094)	Cluster 4 (n = 496)	
Age, years	58.00 (51.00, 64.00)	58.00 (52.00, 65.00)	58.00 (52.00, 64.00)	57.00 (51.00, 63.00)	57.00 (50.00, 62.00)	<0.001
Gender, n (%)						<0.001
Male	2084 (46.06)	695 (50.36)	720 (46.30)	456 (41.68)	213 (42.94)	
Female	2441 (53.94)	685 (49.64)	835 (53.70)	638 (58.32)	283 (57.06)	
Marital status, n (%)						0.1
Married with spouse present	3911 (86.43)	1171 (84.86)	1337 (85.98)	955 (87.29)	448 (90.32)	
Married but not living with spouse temporarily	167 (3.69)	59 (4.28)	54 (3.47)	42 (3.84)	12 (2.42)	
Separated	19 (0.42)	6 (0.43)	7 (0.45)	5 (0.46)	1 (0.20)	
Divorced	22 (0.49)	9 (0.65)	6 (0.39)	4 (0.37)	3 (0.60)	
Widowed	383 (8.46)	122 (8.84)	147 (9.45)	85 (7.77)	29 (5.85)	
Never married	23 (0.51)	13 (0.94)	4 (0.26)	3 (0.27)	3 (0.60)	
Hukou status, n (%)						0.338
Agricultural Hukou	3957 (87.47)	1227 (88.98)	1360 (87.46)	940 (85.92)	430 (86.69)	
Non-Agricultural Hukou	565 (12.49)	152 (11.02)	194 (12.48)	153 (13.99)	66 (13.31)	
Do not have Hukou	2 (0.04)	0 (0.00)	1 (0.06)	1 (0.09)	0 (0.00)	
Educational level, n (%)						0.003
No formal education illiterate	1280 (28.29)	398 (28.86)	438 (28.17)	331 (30.26)	113 (22.78)	
Did not finish primary school but capable of reading or writing	850 (18.79)	281 (20.38)	313 (20.13)	162 (14.81)	94 (18.95)	
Sishu	16 (0.35)	10 (0.73)	3 (0.19)	2 (0.18)	1 (0.20)	
Elementary school	1042 (23.03)	304 (22.04)	351 (22.57)	266 (24.31)	121 (24.40)	
Middle school	941 (20.80)	270 (19.58)	316 (20.32)	228 (20.84)	127 (25.60)	
High school	291 (6.43)	87 (6.31)	100 (6.43)	76 (6.95)	28 (5.65)	
Vocational school	56 (1.24)	12 (0.87)	16 (1.03)	20 (1.83)	8 (1.61)	
Two/Three Year College/Associate degree	42 (0.93)	13 (0.94)	17 (1.09)	8 (0.73)	4 (0.81)	
Four Year College/Bachelor degree	6 (0.13)	4 (0.29)	1 (0.06)	1 (0.09)	0 (0.00)	
Smoking status, n (%)						0.015
Current	1403 (31.01)	478 (34.64)	471 (30.29)	319 (29.16)	135 (27.22)	
Former	333 (7.36)	96 (6.96)	109 (7.01)	92 (8.41)	36 (7.26)	
Never	2789 (61.64)	806 (58.41)	975 (62.70)	683 (62.43)	325 (65.52)	
Drinking status, n (%)						<0.001
Drink more than once a month	1182 (26.12)	437 (31.67)	388 (24.95)	236 (21.57)	121 (24.40)	
Drink but less than once a month	382 (8.44)	120 (8.70)	133 (8.55)	78 (7.13)	51 (10.28)	
Never	2961 (65.44)	823 (59.64)	1034 (66.50)	780 (71.30)	324 (65.32)	
SBP, mmHg	109.00 (98.67, 120.67)	105.33 (96.00, 117.00)	110.00 (98.33, 120.00)	110.33 (100.67, 123.33)	113.00 (104.00, 126.33)	<0.001
DBP, mmHg	74.33 (67.00, 82.33)	72.00 (64.67, 79.33)	74.00 (66.33, 82.00)	75.67 (69.00, 84.33)	78.00 (70.67, 86.67)	<0.001
WC, cm	84.20 (78.00, 91.20)	79.80 (74.20, 85.23)	84.00 (78.00, 90.20)	88.00 (81.53, 94.47)	91.30 (85.00, 97.03)	<0.001
BMI, kg/m^2^	23.09 (20.92, 25.61)	21.52 (19.76, 23.56)	23.03 (21.00, 25.23)	24.33 (22.27, 26.90)	25.45 (23.11, 27.77)	<0.001
Comorbidities						
Hypertension, n (%)						<0.001
Yes	899 (19.98)	187 (13.61)	288 (18.63)	265 (24.31)	159 (32.45)	
No	3601 (80.02)	1187 (86.39)	1258 (81.37)	825 (75.69)	331 (67.55)	
Diabetes, n (%)						<0.001
Yes	196 (4.38)	39 (2.85)	64 (4.15)	55 (5.10)	38 (7.80)	
No	4279 (95.62)	1329 (97.15)	1478 (95.85)	1023 (94.90)	449 (92.20)	
Dyslipidemia, n (%)						<0.001
Yes	315 (7.13)	53 (3.91)	99 (6.53)	101 (9.44)	62 (12.86)	
No	4106 (92.87)	1301 (96.09)	1416 (93.47)	969 (90.56)	420 (87.14)	
MetS, n (%)						<0.001
Yes	1742 (38.50)	114 (8.26)	534 (34.34)	614 (56.12)	480 (96.77)	
No	2783 (61.50)	1266 (91.74)	1021 (65.66)	480 (43.88)	16 (3.23)	
Kidney disease, n (%)						0.522
Yes	242 (5.38)	83 (6.06)	79 (5.12)	58 (5.33)	22 (4.47)	
No	4252 (94.62)	1287 (93.94)	1465 (94.88)	1030 (94.67)	470 (95.53)	
CKD stage, n (%)						0.013
G1	3531 (78.03)	1100 (79.71)	1240 (79.74)	826 (75.50)	365 (73.59)	
G2	945 (20.88)	269 (19.49)	295 (18.97)	258 (23.58)	123 (24.80)	
G3	48 (1.06)	10 (0.72)	20 (1.29)	10 (0.91)	8 (1.61)	
G4	1 (0.02)	1 (0.07)	0 (0.00)	0 (0.00)	0 (0.00)	
CKM syndrome stage, n (%)						<0.001
0	442 (9.77)	271 (19.64)	136 (8.75)	35 (3.20)	0 (0.00)	
1	1108 (24.49)	554 (40.14)	403 (25.92)	151 (13.80)	0 (0.00)	
2	2297 (50.76)	396 (28.70)	804 (51.70)	720 (65.81)	377 (76.01)	
3	678 (14.98)	159 (11.52)	212 (13.63)	188 (17.18)	119 (23.99)	
Medication use						
Hypertension medications, n (%)						0.09
Yes	642 (71.49)	124 (66.31)	201 (69.79)	193 (73.11)	124 (77.99)	
No	256 (28.51)	63 (33.69)	87 (30.21)	71 (26.89)	35 (22.01)	
Diabetes medications, n (%)						0.602
Yes	128 (64.65)	24 (60.00)	40 (60.61)	38 (70.37)	26 (68.42)	
No	70 (35.35)	16 (40.00)	26 (39.39)	16 (29.63)	12 (31.58)	
Dyslipidemia medications, n (%)						0.359
Yes	154 (48.89)	24 (45.28)	45 (45.45)	48 (48.00)	37 (58.73)	
No	161 (51.11)	29 (54.72)	54 (54.55)	52 (52.00)	26 (41.27)	
Laboratory parameters						
WBC, 10^9^/L	5.90 (4.90, 7.20)	5.60 (4.70, 6.70)	5.90 (4.90, 7.12)	6.10 (5.04, 7.40)	6.30 (5.30, 7.70)	<0.001
Hb, g/dL	14.20 (13.00, 15.40)	14.00 (12.90, 15.20)	14.20 (13.00, 15.45)	14.30 (13.10, 15.50)	14.60 (13.20, 15.90)	<0.001
Platelets, 10^9^/L	206.00 (162.00, 254.00)	202.00 (162.00, 251.00)	205.00 (161.00, 255.00)	209.00 (162.00, 255.00)	209.00 (160.00, 261.00)	0.247
FBG, mg/dL	102.24 (94.32, 112.50)	99.54 (92.70, 108.00)	101.70 (94.14, 111.60)	102.78 (95.40, 112.64)	113.40 (102.78, 135.27)	<0.001
HbA1c, %	5.10 (4.90, 5.40)	5.10 (4.80, 5.33)	5.10 (4.90, 5.40)	5.20 (4.90, 5.50)	5.20 (4.90, 5.60)	<0.001
Total cholesterol, mg/dL	190.59 (167.01, 214.95)	185.18 (165.46, 208.38)	187.50 (163.34, 212.24)	197.17 (172.13, 221.91)	199.10 (173.20, 230.80)	<0.001
Triglyceride, mg/dL	104.43 (74.34, 153.10)	64.61 (53.99, 77.22)	107.97 (89.39, 136.29)	137.18 (108.86, 169.92)	287.18 (230.76, 372.58)	<0.001
HDL-C, mg/dL	49.48 (40.59, 60.31)	63.02 (54.90, 73.07)	48.33 (42.14, 55.67)	44.07 (38.66, 50.64)	32.47 (27.84, 36.73)	<0.001
LDL-C, mg/dL	113.66 (93.17, 136.47)	109.21 (91.62, 128.74)	115.21 (95.10, 139.56)	123.33 (102.06, 145.75)	96.65 (71.13, 122.94)	<0.001
CRP, mg/L	0.96 (0.53, 1.97)	0.73 (0.44, 1.57)	0.92 (0.52, 1.87)	1.18 (0.64, 2.33)	1.35 (0.74, 2.55)	<0.001
BUN, mg/dL	15.10 (12.55, 18.12)	15.83 (12.91, 18.91)	14.71 (12.32, 17.58)	15.04 (12.49, 18.03)	15.04 (12.69, 17.74)	<0.001
Scr, mg/dL	0.75 (0.64, 0.87)	0.75 (0.63, 0.86)	0.75 (0.64, 0.86)	0.75 (0.64, 0.87)	0.76 (0.66, 0.88)	0.195
eGFR, mL/min/1.73m^2^	106.99 (92.21, 124.54)	109.25 (93.93, 126.37)	106.96 (93.04, 124.33)	105.75 (90.33, 123.27)	103.40 (89.21, 121.56)	<0.001
AIP_2012_	-0.04 (-0.24, 0.19)	-0.34 (-0.44, -0.25)	-0.02 (-0.11, 0.10)	0.15 (0.01, 0.26)	0.57 (0.47, 0.72)	<0.001
AIP_2015_	-0.01 (-0.18, 0.20)	-0.23 (-0.34, -0.12)	-0.05 (-0.15, 0.03)	0.25 (0.17, 0.37)	0.40 (0.23, 0.58)	<0.001
cumulative AIP	-0.06 (-0.58, 0.52)	-0.10 (-0.35, 0.11)	0.61 (0.44, 0.80)	1.30 (1.10, 1.54)	2.18 (2.18, 2.52)	<0.001
Heart disease, n (%)						0.252
Yes	368 (8.13)	97 (7.03)	141 (9.07)	90 (8.23)	40 (8.06)	
No	4157 (91.87)	1283 (92.97)	1414 (90.93)	1004 (91.77)	456 (91.94)	
Stroke, n (%)						0.006
Yes	246 (5.44)	54 (3.91)	83 (5.34)	75 (6.86)	34 (6.85)	
No	4279 (94.56)	1326 (96.09)	1472 (94.66)	1019 (93.14)	462 (93.15)	
CVD, n (%)						0.04
Yes	578 (12.77)	147 (10.65)	208 (13.38)	153 (13.99)	70 (14.11)	
No	3947 (87.23)	1233 (89.35)	1347 (86.62)	941 (86.01)	426 (85.89)	

SBP, systolic blood pressure; DBP, diastolic blood pressure; WC, waist circumference; BMI, body mass index; MetS, metabolic syndrome; CKD, chronic kidney disease; CKM, Cardiovascular-Kidney-Metabolic; WBC, white blood cell; Hb, hemoglobin; FBG, fasting blood glucose; HbA1c, hemoglobin A1C; HDL-C, high-density lipoprotein cholesterol; LDL-C, low-density lipoprotein cholesterol; CRP, C-reactive protein; BUN, blood urea nitrogen; Scr, serum creatinine; eGFR, estimated glomerular filtration ratio; AIP, atherogenic index of plasma; CVD, cardiovascular disease.

### Association between AIP and study outcomes

Four logistic regression models were developed to evaluate the associations between AIP and CVD incidence among populations across CKM syndrome stages 0-3 (**Table 2**). Compared to cluster 1, the OR (95% CI) for the prevalence of CVD were 1.29 (1.02-1.62) for cluster 2, 1.33 (1.04-1.71) for cluster 3 and 1.35 (0.98-1.85) for cluster 4 after adjusting for certain confounding covariates. Meanwhile, categorizing cumulative AIP into quartiles revealed that compared to the first quartile (Q1), the OR (95% CI) for Q2, Q3, and Q4 were 1.13 (0.87-1.48), 1.37 (1.05-1.77) and 1.33 (1.03-1.74). The trend for P value was 0.014, uncovering a significant association across the quartiles.

**Table 2:** Logistic regression analysis for the association between different classifications and CVD.

	Model I OR (95% CI)	P value	Model II OR (95% CI)	P value	Model III OR (95% CI)	P value	Model IV OR (95% CI)	P value
Change in the AIP								
Cluster 1	Ref.		Ref.		Ref.		Ref.	
Cluster 2	1.30 (1.03-1.62)	0.024	1.30 (1.04-1.63)	0.022	1.29 (1.03-1.62)	0.025	1.29 (1.02-1.62)	0.033
Cluster 3	1.36 (1.07-1.74)	0.012	1.38 (1.08-1.75)	0.01	1.35 (1.05-1.72)	0.018	1.33 (1.04-1.71)	0.024
Cluster 4	1.38 (1.02-1.87)	0.039	1.42 (1.05-1.93)	0.025	1.40 (1.03-1.91)	0.031	1.35 (0.98-1.85)	0.064
Cumulative AIP								
Q1 (<-0.5845)	Ref.		Ref.		Ref.		Ref.	
Q2 (≥-0.5845, <-0.062)	1.17 (0.90-1.52)	0.233	1.17 (0.90-1.51)	0.252	1.17 (0.90-1.52)	0.253	1.13 (0.87-1.48)	0.355
Q3 (≥-0.062, <0.5242)	1.43 (1.11-1.84)	0.005	1.43 (1.11-1.84)	0.006	1.42 (1.10-1.83)	0.007	1.37 (1.05-1.77)	0.018
Q4 (≥0.5242)	1.39 (1.08-1.79)	0.011	1.42 (1.10-1.83)	0.007	1.40 (1.09-1.82)	0.01	1.33 (1.03-1.74)	0.032
p for trend		0.004		0.002		0.004		0.014

CVD, cardiovascular disease; AIP, atherogenic index of plasma; Q, quartile; OR, odds ratio; CI, confidence interval.

Model I was adjusted for none.

Model II was adjusted for age and gender.

Model III was adjusted for age, gender, marital status, Hukou status, educational level, smoking status and drinking status.

Model IV was adjusted for age, gender, marital status, Hukou status, educational level, smoking status, drinking status, white blood cell, hemoglobin, platelets and C-reactive protein.

**Supplementary Tables 1**, **2** presented the associations between AIP and the incidence of the secondary outcomes. No significant relevance was observed between AIP and heart disease in the fully adjusted model (Model IV). Whereas, clusters 2 through 4 displayed a significantly elevated risk of stroke compared to cluster 1. Furthermore, a notable ascending trend in stroke incidence was observed from the first to the fourth quartile of cumulative AIP (P for trend <0.001).

### Dose–response relationship of cumulative AIP and study outcomes

**Figure 2:**
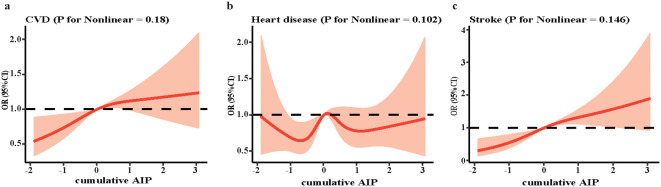
The RCS curve of the association between cumulative AIP and CVD. **(a)** CVD; **(b)** heart disease; **(c)** stroke. RCS regression was adjusted for age, gender, marital status, Hukou status, educational level, smoking status, drinking status, white blood cell, hemoglobin, platelets and C-reactive protein. RCS, restricted cubic spline; AIP, atherogenic index of plasma; CVD, cardiovascular disease; OR, odds ratio; CI, confidence interval.

There was a linearly incremental relationship between the cumulative AIP and the risk of CVD (P for association = 0.006, P for nonlinear = 0.18) and stroke (P for association < 0.001, P for nonlinear = 0.146) by the RCS model. However, the corresponding relationship between cumulative AIP and heart disease was not obvious (P for association = 0.146), consistent with the findings of the regression analysis (**Figure 2**).

### Subgroup analysis of AIP on study outcomes

**Tables 3**, **4** showed the associations between the change in AIP and cumulative AIP with the presence of CVD, stratified by potential risk factors. After adjusting for demographic covariates, the significant interaction was demonstrated in subgroups based on Hukou status and gender.

**Table 3:** Associations of different clusters of change in the AIP with CVD stratified by different factors.

Subgroup	Change in the AIP, OR (95% CI)				P for interaction
	Cluster 1	Cluster 2	Cluster 3	Cluster 4	
Age					0.282
≦60	Ref.	1.50 (1.09, 2.06)*	1.51 (1.08, 2.13)*	1.39 (0.91, 2.11)	
>60	Ref.	1.03 (0.73, 1.45)	1.15 (0.79, 1.67)	1.35 (0.82, 2.19)	
Gender					0.003
Man	Ref.	1.16 (0.80, 1.67)	1.95 (1.33, 2.86)***	1.94 (1.18, 3.14)**	
Female	Ref.	1.40 (1.03, 1.90)*	1.03 (0.74, 1.45)	1.07 (0.70, 1.63)	
Marital status					0.92
Married	Ref.	1.28 (1.01, 1.64)*	1.33 (1.02, 1.73)*	1.32 (0.94, 1.83)	
Others	Ref.	1.36 (0.65, 2.92)	1.65 (0.72, 3.80)	2.04 (0.64, 5.92)	
Hukou status					0.011
Agricultural Hukou	Ref.	1.30 (1.01, 1.67)*	1.52 (1.17, 1.98)**	1.47 (1.04, 2.05)*	
Others	Ref.	1.41 (0.72, 2.79)	0.58 (0.25, 1.33)	0.89 (0.32, 2.31)	
Educational level					0.447
Primary school or lower	Ref.	1.28 (0.98, 1.67)	1.34 (1.00, 1.80)*	1.33 (0.91, 1.93)	
Secondary school or higher	Ref.	1.32 (0.85, 2.09)	1.38 (0.845 2.25)	1.50 (0.82, 2.70)	
Smoking status					0.197
Current	Ref.	1.27 (0.81, 1.99)	1.49 (0.92, 2.41)	2.44 (1.35, 4.33)**	
Former	Ref.	1.31 (0.55, 3.22)	3.16 (1.30, 8.09)*	2.03 (0.53, 7.01)	
Never	Ref.	1.29 (0.96, 1.72)	1.19 (0.87, 1.64)	1.09 (0.72, 1.62)	
Drinking status					0.833
Drink more than once a month	Ref.	1.48 (0.93, 2.38)	1.65 (0.98, 2.79)	1.48 (0.73, 2.87)	
Drink but less than once a month	Ref.	1.26 (0.55, 2.93)	1.84 (0.74, 4.60)	0.87 (0.27, 2.58)	
Never	Ref.	1.23 (0.93, 1.64)	1.21 (0.89, 1.64)	1.35 (0.92, 1.98)	

AIP, atherogenic index of plasma; CVD, cardiovascular disease; OR, odds ratio; CI, confidence interval.

All models were adjusted for age, gender, marital status, Hukou status, educational level, smoking status, drinking status, white blood cell, hemoglobin, platelets and C-reactive protein.

**Table 4:** Associations of the cumulative AIP with CVD stratified by different factors.

Subgroup	Cumulative AIP, OR (95% CI)				P for interaction
	Quartile 1	Quartile 2	Quartile 3	Quartile 4	
Age					0.215
≦60	Ref.	1.40 (0.97, 2.03)	1.67 (1.17, 2.40)**	1.39 (0.97, 2.00)	
>60	Ref.	0.89 (0.60, 1.32)	1.09 (0.74, 1.60)	1.32 (0.90, 1.95)	
Gender					0.03
Man	Ref.	1.06 (0.69, 1.62)	1.56 (1.04, 2.34)*	1.97 (1.33, 2.94)***	
Female	Ref.	1.16 (0.82, 1.65)	1.25 (0.88, 1.77)	1.01 (0.70, 1.44)	
Marital status					0.151
Married	Ref.	1.09 (0.82, 1.45)	1.45 (1.10, 1.90)**	1.31 (1.00, 1.73)	
Others	Ref.	1.58 (0.72, 3.61)	0.87 (0.34, 2.17)	1.75 (0.73, 4.23)	
Hukou status					0.037
Agricultural Hukou	Ref.	1.24 (0.93, 1.64)	1.45 (1.09, 1.92)**	1.56 (1.178, 2.07)**	
Others	Ref.	0.63 (0.26, 1.45)	1.19 (0.58, 2.46)	0.56 (0.26, 1.234)	
Educational level					0.992
Primary school or lower	Ref.	1.05 (0.77, 1.44)	1.35 (1.00, 1.82)	1.32 (0.97, 1.79)	
Secondary school or higher	Ref.	1.47 (0.87, 2.50)	1.47 (0.88, 2.48)	1.50 (0.90, 2.53)	
Smoking status					0.593
Current	Ref.	1.14 (0.68, 1.91)	1.78 (1.10, 2.90)*	1.57 (0.96, 2.60)	
Former	Ref.	1.65 (0.65, 4.30)	1.56 (0.58, 4.20)	2.86 (1.16, 7.41)*	
Never	Ref.	1.07 (0.77, 1.50)	1.23 (0.89, 1.72)	1.18 (0.84, 1.65)	
Drinking status					0.399
Drink more than once a month	Ref.	1.48 (0.88, 2.49)	1.63 (0.95, 2.81)	1.64 (0.97, 2.80)	
Drink but less than once a month	Ref.	1.06 (0.43, 2.64)	0.79 (0.29, 2.06)	1.80 (0.76, 4.43)	
Never	Ref.	1.01 (0.73, 1.41)	1.34 (0.98, 1.84)	1.16 (0.84, 1.61)	

AIP, atherogenic index of plasma; CVD, cardiovascular disease; OR, odds ratio; CI, confidence interval.

All models were adjusted for age, gender, marital status, Hukou status, educational level, smoking status, drinking status, white blood cell, hemoglobin, platelets and C-reactive protein.

With respect to heart disease, no interaction was found between change in AIP clusters or cumulative AIP and subgroup variables (**Supplementary Tables 3**, **4**). Except for Hukou status, no significant interactions were observed across subgroups for stroke (P for interaction >0.05) (**Supplementary Tables 5**, **6**).

### WQS analyses

WQS regression models were utilized to thoroughly elucidate the risk factors for CVD. As illustrated in **Supplementary Figure 2**, TG accounted for a large proportion of the occurrence of both the primary and secondary outcomes.

## Discussion

To explore the association between AIP and CVD among older individuals with CKM syndrome stages 0–3, we performed a longitudinal study involving 4,525 participants from the CHARLS study. Our study confirmed that a chronically elevated AIP or a superior cumulative AIP was associated with a elevated incidence of CVD and stroke events, but no such association was observed with heart disease. Notably, TG was identified as the dominant contributing factor to CVD. The above results underscored that AIP level was a robust predictor of CVD risk among populations with CKM syndrome stages 0 to 3.

CKM syndrome typically arises from excess or dysfunctional adipose tissue (4), which accelerates atherosclerosis by exacerbating inflammation, lipid abnormalities, abnormal blood pressure, and insulin resistance (26). Thus, CKM syndrome represents the interconnected pathophysiological effects of metabolic risk factors of obesity, DM, CKD, and CVD. AIP was a composite parameter relevant to aberrant glucose and lipid metabolism (27), firstly proposed by Dobiásová and Frohlich (7). Zhu et al. affirmed that AIP was a positive and strong predictor of obesity (28). Besides, AIP has been linked to an increased risk of diabetes across various populations (10, 11), as well as prehypertension or hypertension (8, 29). A comprehensive review has further summarized that AIP may be associated with multiple cardiometabolic risk factors (30). Hu et al. indicated that AIP was positively correlated with CVD risk in Chinese individuals in CKM stages 0-3 (31). Zhang et al. demonstrated the prognostic values of the incidence of CVD by cumulative AIP and AIP control in individuals with CKM syndrome from stages 1 to 3 (32). Collectively, these studies have proved that AIP serves as a potent predictor of prognosis in CKM syndrome.

Min et al. revealed that constantly higher AIP may have a higher incidence of CVD in hyperglycemic individuals (17). In this study, the population was split into disparate clusters according to the dynamic changes of AIP levels from 2012 to 2015. Cluster 1 represented a consistently low AIP, as the control group; cluster 2 showed a moderate AIP with a descending trend; cluster 3 displayed a moderate AIP with a rising trend; and cluster 4 maintained the highest AIP despite a declining trend. The results indicated that when AIP remained within a high range - regardless of whether it increased or decreased - the risk of CVD events showed no improvement. In contrast, maintaining AIP at very low levels was related to a potentially dramatical reduction in the incidence of CVD. However, with regard to the secondary outcomes, no impact of AIP change on the cause of heart disease was observed, which was not consistent with findings from previous literature (19, 33, 34). A mass of studies primarily paid close attention to AIP measurements at a single time point, and failed to investigate the impact of dynamic fluctuations in AIP on the occurrence of CVD. This discrepancy highlights the need for further prospective trials to validate these divergent results in the future.

It has been reported that AIP was regarded as an independent predictor of CVD events (29), with the underlying mechanism attributed to its correlation with the size of lipoprotein particle (35). With the increase of AIP levels, the incidence of CVD presented a corresponding upward trend (35). Our study also throwed the latent relation between cumulative AIP and both primary and secondary outcomes. Specifically, there was a linear relationship between cumulative AIP and the risk of CVD and stroke. Thus, the interventions aimed at controlling AIP levels are crucial for reducing CVD risk.

The effect of Hukou status on the occurrence of CVD was showed in the subgroup analysis of this study. Our project confirmed that the availability of AIP in monitoring CVD was more potent in rural areas as a whole. Possible reasons may have contributed to this observation. First, there were shortage of medical institutions and healthcare professionals, making it difficult for rural residents to access timely diagnosis, treatment and preventive services. Second, the limited income of rural residents may hinder their ability to afford a healthy diet, regular medical check-ups, and necessary medications. Third, urbanization has contributed to the migration of young labors from rural areas, exacerbating the aging population in these regions.

At present, the most well-established risk factors for CVD events include lipoprotein and glucose metabolism disorders (36). In order to thoroughly investigate the contributions of these metabolic disorders to CVD, the WQS analyses were adapted to assess the relative proportion of TG, TC, HDL-C, LDL-C and FBG. Both the primary and secondary outcomes identified TG as a primary biochemical indicator contributing to CVD events.

Our study enhances the existing knowledge hierarchy by affording evidence in favor of the the utility of dynamic AIP changes as a valuable biologic-indicator for discerning individuals at high risk for CVD.

AIP, stemmed from the calculation of TG and HDL-C, can be conveniently measured from a single blood sample, offering a cost-effective and convenient alternative for risk assessment. Therefore, AIP holds significant promise as a promising potential marker for picking out patients at increased risk for CVD, particularly for stroke.

However, several limitations must be acknowledged in our study. First, our participants were exclusively drawn from the Chinese middle-aged and elderly populations, which may limit the applicability of our findings to other countries or age groups. Second, the participants self-reported CVD status, which may introduce over-reporting or under-reporting bias. Third, despite employing multivariate adjustments and conducting subgroup analyses, the study was a single-center research and some latent confounding covariates may have been overlooked.

## Conclusions

In conclusion, we emphasized that significant changes in the AIP are independently related to the increased risk of CVD in Chinese individuals (age <45) with CKM syndrome stage 0-3. Consequently, supervising long-term fluctuations in AIP should be prioritized in CVD prevention strategies. Furthermore, our findings highlighted that TG was the dominating contributor to the gross effects. Moving forward, AIP holds promise as a precious appliance for assessing CVD risk in this group, but intensive studies are warranted to conduct in-depth analyses and explore its broader applicability.

## Data Availability

The datasets presented in this study can be found in online repositories. The names of the repository/repositories and accession number(s) can be found below: https://charls.pku.edu.cn/.
