# Supplementary material for: Association between atherogenic index of plasma and future cardiovascular disease risk in middle-aged and elderly individuals with cardiovascular-kidney-metabolic syndrome stage 0-3
Source: Front Endocrinol (Lausanne). 2025 Mar 14;16:1540241. doi: 10.3389/fendo.2025.1540241 (PMC11949822; doi:10.3389/fendo.2025.1540241)
Supplement: Supplementary file 1 [file DataSheet1.docx]

**

**

Supplementary figure 1: Flow chat of the study population. CKM, cardiovascular-kidney-metabolic; TG, triglyceride; HDL-C, high-density lipoprotein cholesterol; CVD, cardiovascular disease.

**
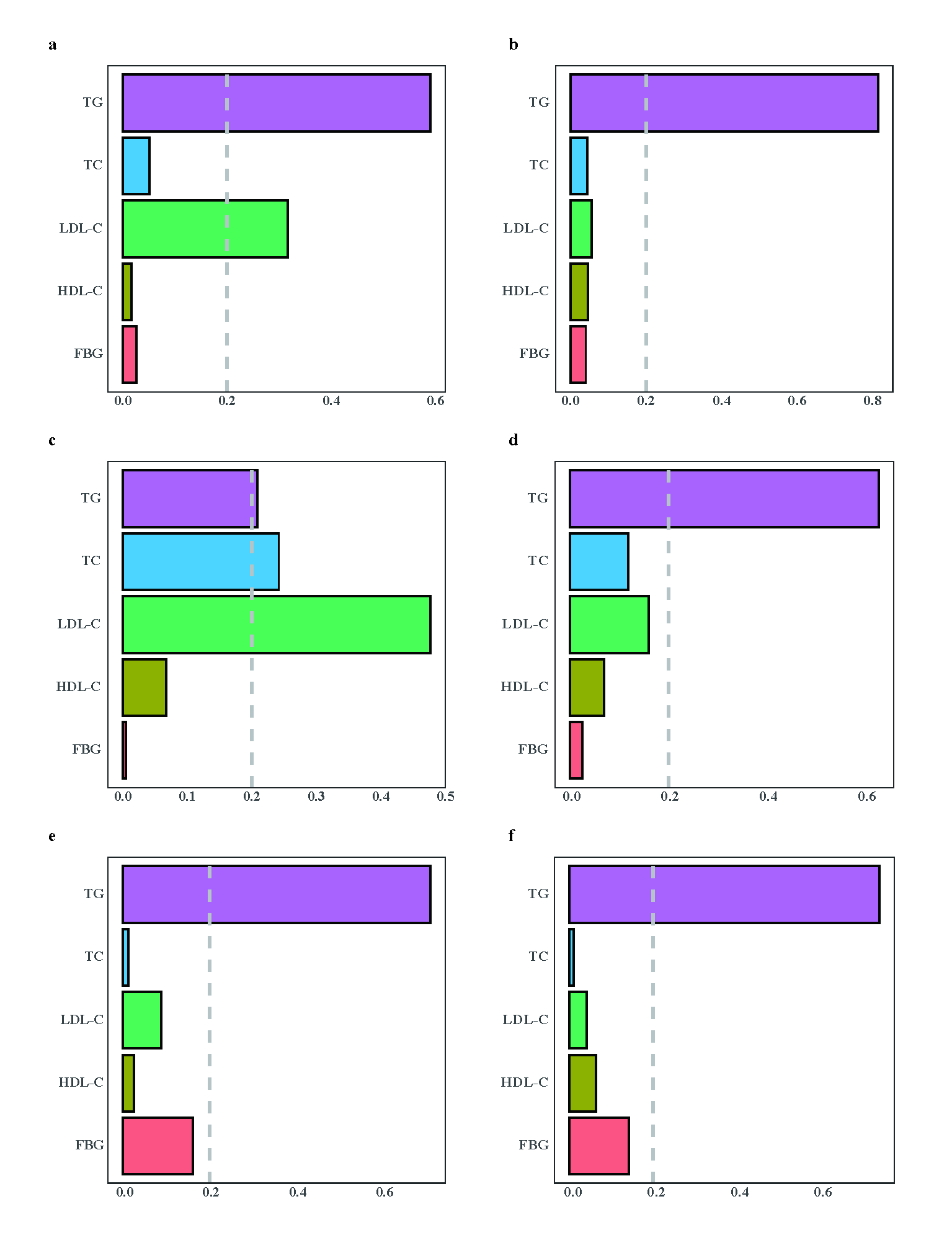
**

**Supplementary figure 2:** Estimated weights assigned to AIP with the WQS model. (a) Weights for CVD in 2012; (b) Weights for CVD in 2015; (c) Weights for heart disease in 2012; (d) Weights for heart disease in 2015; (e) Weights for stroke in 2012. (f) Weights for stroke in 2015. WQS models were adjusted for age, gender, marital status, Hukou status, educational level, smoking status, drinking status, white blood cell, hemoglobin, platelets and C-reactive protein. WQS, weighted quantile sum; AIP, atherogenic index of plasma; CVD, cardiovascular disease; TG, triglyceride; TC, total cholesterol; LDL-C, low-density lipoprotein cholesterol; HDL-C, high-density lipoprotein cholesterol; FBG, fasting blood glucose.

**Supplementary table 1 Logistic regression analysis for the association between different classifications and heart disease.**

|  | Model I OR (95% CI) | P value | Model II  OR (95% CI) | P value | Model III  OR (95% CI) | P value | Model IV  OR (95% CI) | P value |
| --- | --- | --- | --- | --- | --- | --- | --- | --- |
| Change in the AIP | | | | | | | | |
| Cluster 1 | Ref. |  | Ref. |  | Ref. |  | Ref. |  |
| Cluster 2 | 1.32 (1.01-1.73) | 0.044 | 1.30 (0.99-1.70) | 0.057 | 1.30 (0.99-1.70) | 0.06 | 1.29 (0.98-1.70) | 0.07 |
| Cluster 3 | 1.19 (0.88-1.60) | 0.264 | 1.15 (0.85-1.55) | 0.36 | 1.12 (0.83-1.52) | 0.455 | 1.11 (0.82-1.51) | 0.499 |
| Cluster 4 | 1.16 (0.79-1.70) | 0.448 | 1.14 (0.78-1.68) | 0.499 | 1.12 (0.76-1.64) | 0.574 | 1.08 (0.73-1.59) | 0.712 |
| Cumulative AIP | | | | | | | | |
| Q1 (<-0.5845) | Ref. |  | Ref. |  | Ref. |  | Ref. |  |
| Q2 (≥-0.5845, <-0.062) | 1.17 (0.85-1.60) | 0.336 | 1.13 (0.82-1.55) | 0.456 | 1.14 (0.83-1.56) | 0.425 | 1.09 (0.79-1.50) | 0.605 |
| Q3 (≥-0.062, <0.5242) | 1.45 (1.07-1.97) | 0.015 | 1.40 (1.03-1.89) | 0.031 | 1.40 (1.03-1.90) | 0.033 | 1.33 (0.98-1.82) | 0.069 |
| Q4 (≥0.5242) | 1.17 (0.85-1.60) | 0.336 | 1.14 (0.83-1.56) | 0.431 | 1.11 (0.81-1.53) | 0.519 | 1.06 (0.77-1.47) | 0.706 |
| p for trend |  | 0.174 |  | 0.238 |  | 0.288 |  | 0.455 |

Abbreviations: AIP, atherogenic index of plasma; Q, quartile; OR, odds ratio; CI, confidence interval.

Model I was adjusted for none.

Model II was adjusted for age and gender.

Model III was adjusted for age, gender, marital status, Hukou status, educational level, smoking status and drinking status.

Model IV was adjusted for age, gender, marital status, Hukou status, educational level, smoking status, drinking status, white blood cell, hemoglobin, platelets and C-reactive protein.

**Supplementary table 2 Logistic regression analysis for the association between different classifications and stroke.**

|  | Model I OR (95% CI) | P value | Model II  OR (95% CI) | P value | Model III  OR (95% CI) | P value | Model IV  OR (95% CI) | P value |
| --- | --- | --- | --- | --- | --- | --- | --- | --- |
| Change in the AIP | | | | | | | | |
| Cluster 1 | Ref. |  | Ref. |  | Ref. |  | Ref. |  |
| Cluster 2 | 1.38 (0.98-1.97) | 0.069 | 1.45 (1.02-2.06) | 0.04 | 1.43 (1.01-2.04) | 0.046 | 1.43 (1.00-2.06) | 0.051 |
| Cluster 3 | 1.81 (1.26-2.59) | 0.001 | 1.96 (1.36-2.82) | <0.001 | 1.93 (1.34-2.78) | <0.001 | 1.92 (1.32-2.79) | <0.001 |
| Cluster 4 | 1.81 (1.16-2.81) | 0.009 | 2.05 (1.31-3.20) | 0.002 | 2.04 (1.30-3.20) | 0.002 | 1.96 (1.24-3.10) | 0.004 |
| Cumulative AIP | | | | | | | | |
| Q1 (<-0.5845) | Ref. |  | Ref. |  | Ref. |  | Ref. |  |
| Q2 (≥-0.5845, <-0.062) | 1.29 (0.86-1.93) | 0.221 | 1.35 (0.90-2.02) | 0.149 | 1.33 (0.88-2.00) | 0.17 | 1.34 (0.89-2.04) | 0.164 |
| Q3 (≥-0.062, <0.5242) | 1.48 (1.00-2.19) | 0.05 | 1.58 (1.06-2.35) | 0.023 | 1.57 (1.05-2.34) | 0.027 | 1.55 (1.03-2.34) | 0.035 |
| Q4 (≥0.5242) | 1.93 (1.33-2.81) | <0.001 | 2.16 (1.48-3.16) | <0.001 | 2.14 (1.46-3.13) | <0.001 | 2.07 (1.39-3.07) | <0.001 |
| p for trend |  | <0.001 |  | <0.001 |  | <0.001 |  | <0.001 |

Abbreviations: AIP, atherogenic index of plasma; Q, quartile; OR, odds ratio; CI, confidence interval.

Model I was adjusted for none.

Model II was adjusted for age and gender.

Model III was adjusted for age, gender, marital status, Hukou status, educational level, smoking status and drinking status.

Model IV was adjusted for age, gender, marital status, Hukou status, educational level, smoking status, drinking status, white blood cell, hemoglobin, platelets and C-reactive protein.

**Supplementary table 3 Associations of different clusters of change in the AIP with heart disease stratified by different factors.**

| Subgroup | Change in the AIP, OR (95% CI) | | | | P for interaction |
| --- | --- | --- | --- | --- | --- |
|  | Cluster 1 | Cluster 2 | Cluster 3 | Cluster 4 |  |
| Age |  |  |  |  | 0.39 |
| ≦60 | Ref. | 1.49 (1.04, 2.17)* | 1.33 (0.89, 1.98) | 1.06 (0.62, 1.77) |  |
| >60 | Ref. | 1.06 (0.69, 1.61) | 0.88 (0.54, 1.43) | 1.20 (0.64, 2.17) |  |
| Gender |  |  |  |  | 0.17 |
| Man | Ref. | 1.16 (0.73, 1.86) | 1.55 (0.93, 2.56) | 1.55 (0.79, 2.92) |  |
| Female | Ref. | 1.39 (0.98, 1.98) | 0.94 (0.63, 1.40) | 0.93 (0.55, 1.52) |  |
| Marital status |  |  |  |  | 0.191 |
| Married | Ref. | 1.25 (0.94, 1.66) | 1.15 (0.84, 1.58) | 1.06 (0.70, 1.58) |  |
| Others | Ref. | 2.48 (0.89, 7.72) | 0.66 (0.09, 3.13) | 2.21 (0.29, 11.60) |  |
| Hukou status |  |  |  |  | 0.088 |
| Agricultural Hukou | Ref. | 1.30 (0.97, 1.75) | 1.26 (0.91, 1.75) | 1.09 (0.70, 1.65) |  |
| Others | Ref. | 1.43 (0.68, 3.11) | 0.58 (0.21, 1.50) | 1.26 (0.42, 3.50) |  |
| Educational level |  |  |  |  | 0.798 |
| Primary school or lower | Ref. | 1.26 (0.91, 1.74) | 1.13 (0.79, 1.62) | 1.09 (0.68, 1.72) |  |
| Secondary school or higher | Ref. | 1.39 (0.82, 2.39) | 1.10 (0.60, 2.01) | 1.11 (0.51, 2.31) |  |
| Smoking status |  |  |  |  | 0.581 |
| Current | Ref. | 1.09 (0.63, 1.92) | 1.07 (0.57, 1.97) | 1.86 (0.87, 3.81) |  |
| Former | Ref. | 1.43 (0.52, 4.03) | 1.09 (0.35, 3.35) | 0.65 (0.09, 3.05) |  |
| Never | Ref. | 1.38 (0.99, 1.95) | 1.21 (0.83, 1.76) | 0.99 (0.59, 1.60) |  |
| Drinking status |  |  |  |  | 0.961 |
| Drink more than once a month | Ref. | 1.27 (0.74, 2.20) | 0.88 (0.43, 1.70) | 0.99 (0.40, 2.23) |  |
| Drink but less than once a month | Ref. | 1.69 (0.62, 4.89) | 2.34 (0.75, 7.54) | 1.44 (0.34, 5.38) |  |
| Never | Ref. | 1.27 (0.91, 1.79) | 1.12 (0.78, 1.62) | 1.09 (0.67, 1.74) |  |

Abbreviations: AIP, atherogenic index of plasma; OR, odds ratio; CI, confidence interval.

All models were adjusted for age, gender, marital status, Hukou status, educational level, smoking status, drinking status, white blood cell, hemoglobin, platelets and C-reactive protein.

**Supplementary table 4 Associations of the cumulative AIP with heart disease stratified by different factors.**

| Subgroup | Cumulative AIP, OR (95% CI) | | | | P for interaction |
| --- | --- | --- | --- | --- | --- |
|  | Quartile 1 | Quartile 2 | Quartile 3 | Quartile 4 |  |
| Age |  |  |  |  | 0.485 |
| ≦60 | Ref. | 1.44 (0.95, 2.23) | 1.60 (1.06, 2.45)* | 1.17 (0.75, 1.82) |  |
| >60 | Ref. | 0.74 (0.45, 1.22) | 1.07 (0.67, 1.71) | 1.01 (0.62, 1.66) |  |
| Gender |  |  |  |  | 0.256 |
| Man | Ref. | 0.93 (0.53, 1.61) | 1.70 (1.03, 2.82)* | 1.43 (0.84, 2.44) |  |
| Female | Ref. | 1.15 (0.77, 1.72) | 1.19 (0.80, 1.77) | 0.90 (0.60, 1.38) |  |
| Marital status |  |  |  |  | 0.399 |
| Married | Ref. | 1.05 (0.75, 1.46) | 1.36 (0.99, 1.87) | 1.08 (0.77, 1.50) |  |
| Others | Ref. | 1.98 (0.63, 6.90) | 1.45 (0.39, 5.59) | 0.96 (0.18, 4.34) |  |
| Hukou status |  |  |  |  | 0.41 |
| Agricultural Hukou | Ref. | 1.14 (0.81, 1.61) | 1.39 (0.99, 1.94) | 1.19 (0.84, 1.69) |  |
| Others | Ref. | 0.91 (0.35, 2.35) | 1.31 (0.57, 3.11) | 0.64 (0.25, 1.62) |  |
| Educational level |  |  |  |  | 0.984 |
| Primary school or lower | Ref. | 1.01 (0.70, 1.47) | 1.32 (0.92, 1.90) | 1.07 (0.74, 1.57) |  |
| Secondary school or higher | Ref. | 1.41 (0.76, 2.65) | 1.40 (0.77, 2.60) | 1.10 (0.58, 2.10) |  |
| Smoking status |  |  |  |  | 0.936 |
| Current | Ref. | 0.99 (0.51, 1.92) | 1.59 (0.87, 2.94) | 1.13 (0.58, 2.17) |  |
| Former | Ref. | 1.45 (0.47, 4.56) | 1.18 (0.34, 3.99) | 1.02 (0.32, 3.28) |  |
| Never | Ref. | 1.10 (0.74, 1.64) | 1.32 (0.90, 1.95) | 1.09 (0.73, 1.64) |  |
| Drinking status |  |  |  |  | 0.486 |
| Drink more than once a month | Ref. | 1.50 (0.82, 2.78) | 1.25 (0.64, 2.44) | 1.08 (0.54, 2.13) |  |
| Drink but less than once a month | Ref. | 0.86 (0.26, 2.79) | 1.14 (0.36, 3.59) | 1.92 (0.65, 5.94) |  |
| Never | Ref. | 0.97 (0.65, 1.44) | 1.36 (0.94, 1.99) | 0.97 (0.65, 1.45) |  |

Abbreviations: AIP, atherogenic index of plasma; OR, odds ratio; CI, confidence interval.

All models were adjusted for age, gender, marital status, Hukou status, educational level, smoking status, drinking status, white blood cell, hemoglobin, platelets and C-reactive protein.

**Supplementary table 5 Associations of different clusters of change in the AIP with stroke stratified by different factors.**

| Subgroup | Change in the AIP, OR (95% CI) | | | | P for interaction |
| --- | --- | --- | --- | --- | --- |
|  | Cluster 1 | Cluster 2 | Cluster 3 | Cluster 4 |  |
| Age |  |  |  |  | 0.129 |
| ≦60 | Ref. | 1.85 (1.08, 3.30)* | 2.09 (1.19, 3.80)* | 2.58 (1.35, 4.96)** |  |
| >60 | Ref. | 1.14 (0.70, 1.86) | 1.83 (1.12, 3.02)* | 1.43 (0.69, 2.82) |  |
| Gender |  |  |  |  | 0.09 |
| Man | Ref. | 1.28 (0.76, 2.17) | 2.56 (1.54, 4.33)*** | 2.41 (1.23, 4.62)** |  |
| Female | Ref. | 1.56 (0.94, 2.66) | 1.41 (0.81, 2.47) | 1.56 (0.79, 3.00) |  |
| Marital status |  |  |  |  | 0.682 |
| Married | Ref. | 1.59 (1.07, 2.39)* | 1.98 (1.32, 3.02)** | 2.05 (1.24, 3.37)** |  |
| Others | Ref. | 0.82 (0.33, 2.04) | 1.81 (0.74, 4.55) | 1.51 (0.38, 5.15) |  |
| Hukou status |  |  |  |  | 0.036 |
| Agricultural Hukou | Ref. | 1.42 (0.96, 2.11) | 2.14 (1.45, 3.19)*** | 2.31 (1.42, 3.72)*** |  |
| Others | Ref. | 1.61 (0.59, 4.79) | 0.92 (0.25, 3.26) | 0.41 (0.02, 2.80) |  |
| Educational level |  |  |  |  | 0.763 |
| Primary school or lower | Ref. | 1.41 (0.93, 2.14) | 1.84 (1.20, 2.84)** | 1.79 (1.01, 3.09)* |  |
| Secondary school or higher | Ref. | 1.51 (0.73, 3.30) | 2.26 (1.08, 4.97)* | 2.81 (1.17, 6.74)* |  |
| Smoking status |  |  |  |  | 0.179 |
| Current | Ref. | 1.96 (1.03, 3.85)* | 2.62 (1.34, 5.27)** | 3.42 (1.48, 7.76)** |  |
| Former | Ref. | 1.24 (0.37, 4.24) | 5.90 (1.97, 20.32)** | 3.91 (0.83, 17.43) |  |
| Never | Ref. | 1.23 (0.78, 1.99) | 1.34 (0.81, 2.23) | 1.40 (0.75, 2.56) |  |
| Drinking status |  |  |  |  | 0.119 |
| Drink more than once a month | Ref. | 2.31 (1.11, 5.08)* | 4.31 (2.07, 9.49)*** | 2.67 (0.95, 7.14) |  |
| Drink but less than once a month | Ref. | 0.64 (0.86, 2.13) | 1.09 (0.95, 2.45) | 0.81 (0.16, 3.33) |  |
| Never | Ref. | 1.35 (0.86, 2.13) | 1.52 (0.95, 2.45) | 1.93 (1.09, 3.39)* |  |

Abbreviations: AIP, atherogenic index of plasma; OR, odds ratio; CI, confidence interval.

All models were adjusted for age, gender, marital status, Hukou status, educational level, smoking status, drinking status, white blood cell, hemoglobin, platelets and C-reactive protein.

**Supplementary table 6 Associations of the cumulative AIP with stroke stratified by different factors.**

| Subgroup | Cumulative AIP, OR (95% CI) | | | | P for interaction |
| --- | --- | --- | --- | --- | --- |
|  | Quartile 1 | Quartile 2 | Quartile 3 | Quartile 4 |  |
| Age |  |  |  |  | 0.725 |
| ≦60 | Ref. | 1.57 (0.84, 3.02) | 1.91 (1.04, 3.63)* | 2.21 (1.24, 4.13)** |  |
| >60 | Ref. | 1.20 (0.69, 2.10) | 1.33 (0.76, 2.33) | 2.01 (1.18, 3.48)* |  |
| Gender |  |  |  |  | 0.249 |
| Man | Ref. | 1.31 (0.73, 2.36) | 1.49 (0.84, 2.68) | 2.73 (1.61, 4.73)*** |  |
| Female | Ref. | 1.40 (0.77, 2.62) | 1.57 (0.87, 2.91) | 1.53 (0.85, 2.83) |  |
| Marital status |  |  |  |  | 0.201 |
| Married | Ref. | 1.42 (0.90, 2.28) | 1.85 (1.19, 2.93)** | 2.21 (1.44, 3.46)*** |  |
| Others | Ref. | 1.04 (0.40, 2.79) | 0.66 (0.22, 1.92) | 1.79 (0.69, 4.83) |  |
| Hukou status |  |  |  |  | 0.013 |
| Agricultural Hukou | Ref. | 1.55 (1.00, 2.42) | 1.58 (1.01, 2.49)* | 2.50 (1.65, 3.86)*** |  |
| Others | Ref. | 0.30 (0.04, 1.44) | 1.65 (0.57, 5.14) | 0.56 (0.14, 2.09) |  |
| Educational level |  |  |  |  | 0.985 |
| Primary school or lower | Ref. | 1.22 (0.76, 1.97) | 1.55 (0.98, 2.48) | 1.92 (1.22, 3.06)** |  |
| Secondary school or higher | Ref. | 1.97 (0.84, 4.87) | 1.71 (0.73, 4.26) | 3.03 (1.38, 7.23)** |  |
| Smoking status |  |  |  |  | 0.483 |
| Current | Ref. | 1.59 (0.75, 3.43) | 2.43 (1.21, 5.10)* | 2.7 (1.35, 5.64)** |  |
| Former | Ref. | 1.58 (0.43, 6.10) | 2.15 (0.60, 8.23) | 5.59 (1.77, 20.67)** |  |
| Never | Ref. | 1.18 (0.69, 2.04) | 1.15 (0.67, 2.00) | 1.54 (0.92, 2.65) |  |
| Drinking status |  |  |  |  | 0.224 |
| Drink more than once a month | Ref. | 1.43 (0.61, 3.39) | 2.88 (1.31, 6.57)** | 3.44 (1.63, 7.67)** |  |
| Drink but less than once a month | Ref. | 1.24 (0.34, 4.77) | 0.39 (0.05, 2.01) | 1.89 (0.54, 7.37) |  |
| Never | Ref. | 1.31 (0.78, 2.23) | 1.40 (0.85, 2.36) | 1.70 (1.04, 2.85)* |  |

Abbreviations: AIP, atherogenic index of plasma; OR, odds ratio; CI, confidence interval.

All models were adjusted for age, gender, marital status, Hukou status, educational level, smoking status, drinking status, white blood cell, hemoglobin, platelets and C-reactive protein.
